# Arrays of Plasmonic Nanostructures for Absorption Enhancement in Perovskite Thin Films

**DOI:** 10.3390/nano10071342

**Published:** 2020-07-09

**Authors:** Tianyi Shen, Qiwen Tan, Zhenghong Dai, Nitin P. Padture, Domenico Pacifici

**Affiliations:** School of Engineering, Brown University, 184 Hope Street, Providence, RI 02912, USA; tianyi_shen@brown.edu (T.S.); qiwen_tan@brown.edu (Q.T.); zhenghong_dai@brown.edu (Z.D.); nitin_padture@brown.edu (N.P.P.)

**Keywords:** perovskite solar cells, surface plasmon polaritons, plasmonic nanostructures, absorption enhancement, FDTD simulations

## Abstract

We report optical characterization and theoretical simulation of plasmon enhanced methylammonium lead iodide (MAPbI${}_{3}$) thin-film perovskite solar cells. Specifically, various nanohole (NH) and nanodisk (ND) arrays are fabricated on gold/MAPbI${}_{3}$ interfaces. Significant absorption enhancement is observed experimentally in 75 nm and 110 nm-thick perovskite films. As a result of increased light scattering by plasmonic concentrators, the original Fabry–Pérot thin-film cavity effects are suppressed in specific structures. However, thanks to field enhancement caused by plasmonic resonances and in-plane interference of propagating surface plasmon polaritons, the calculated overall power conversion efficiency (PCE) of the solar cell is expected to increase by up to 45.5%, compared to its flat counterpart. The role of different geometry parameters of the nanostructure arrays is further investigated using three dimensional (3D) finite-difference time-domain (FDTD) simulations, which makes it possible to identify the physical origin of the absorption enhancement as a function of wavelength and design parameters. These findings demonstrate the potential of plasmonic nanostructures in further enhancing the performance of photovoltaic devices based on thin-film perovskites.

## 1. Introduction

Hybrid organic–inorganic perovskites have become one of the most popular photovoltaic materials due to their high absorption coefficient [1], long carrier diffusion length [2] as well as low-cost fabrication process [3,4,5,6,7], which make them excellent candidates for high-efficiency solar cells. As one of the most popular members of perovskite materials, methylammonium lead iodide or CH${}_{3}$NH${}_{3}$PbI${}_{3}$ (MAPbI${}_{3}$) possesses such a high absorption coefficient that a 400 nm-thick film is sufficient to absorb most of the incident solar spectrum below its bandgap. Although the record power conversion efficiency (PCE) of a single-junction perovskite solar cell has reached values up to 25.2% [8], there is still much room to boost the PCE. Therefore, several methods have been proposed to improve the performance of perovskite solar cells, one of which involves the use of surface plasmons. Plasmonic effects could be employed to improve the performance of perovskite solar cells [9,10,11,12,13,14,15] by embedding nanoparticles [16,17,18,19,20,21,22,23,24,25,26,27,28,29,30] or plasmonic concentrators [31,32,33,34,35] that can increase absorption, especially near the optical band edge of the material. Previous theoretical research has shown that nanohole (NH) or nanodisk (ND) arrays embedded on gold electrodes with a thin layer of MAPbI${}_{3}$ deposited on them can significantly improve the solar cell PCE by up to ∼10%. The reduced film thickness has the implied benefits of reducing the amount of toxic materials, improving electronic performance and enabling fabrication on flexible substrates [36].

In this work, we report the experimental fabrication, structural and optical characterization, as well as theoretical simulations of plasmon enhanced MAPbI${}_{3}$ perovskite solar cell with NH and ND arrays on gold substrates, as a function of geometry parameters and varying thickness of MAPbI${}_{3}$ deposited on top. Four geometry features, height *h*, diameter *D*, pitch *P* and MAPbI${}_{3}$ thickness *t*, are systematically studied by three-dimensional finite-difference time-domain (3D FDTD) simulations to identify the physical effects responsible for the absorption and PCE enhancement.

## 2. Materials and Methods

An adhesion layer of 4 nm-thick titanium and a layer of 200 nm-thick gold was sequentially deposited on a 1 mm-thick quartz substrate using electron beam evaporation. Subsequently, NH or ND arrays of varying geometry parameters were fabricated on the thick gold film using focused ion beam (FIB) milling.

**Figure 1:**
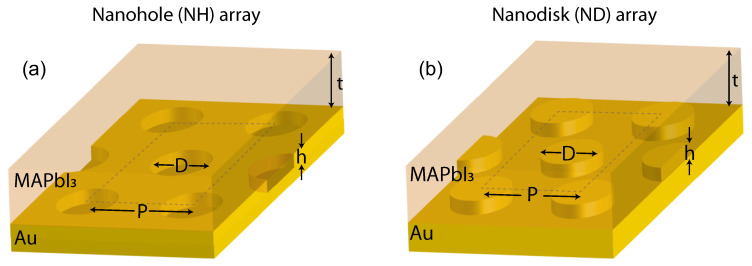
Schematic illustrations of methylammonium lead iodide (MAPbI${}_{3}$) thin-film over nanostructured gold (Au) surface with (**a**) NH or (**b**) ND array, respectively. *t* represents the perovskite film thickness on the top of gold surface. *P* represents the triangular array pitch, while *h* and *D* respectively represent the height and diameter of a plasmonic concentrator. A negative *h* indicated NH, while a positive *h* corresponded to ND.

Following the FIB milling, the substrates were treated with UV-ozone for 45 min to enhance the wettability. The MAPbI${}_{3}$ precursor solution was prepared by dissolving 159 mg of methylammonium iodide (Greatcell, Queanbeyan, Australia) and 461 mg of PbI${}_{2}$ (Sigma-Aldrich, St. Louis, MO, USA) in 78 mg dimethyl sulfoxide (Sigma-Aldrich, St. Louis, MO, USA) and 1368 mg of N,N-dimethylformamide (Acros organics, NJ, USA) to obtain a 30 wt% solution. To deposit the MAPbI${}_{3}$ layer, the solution was spin-coated at 4000 rpm for 30 s with an acceleration of 1300 rpm/s in a nitrogen-filled glove box. At the 10th second of spinning, 250 μL diethyl ether (Sigma-Aldrich, St. Louis, MO, USA) was dripped onto the substrate. The as-coated film was then annealed at 100 ${}^{\circ }$C for 20 min to obtain the MAPbI${}_{3}$ thin film. The film thickness can be controlled by adjusting the amount of N,N-dimethylformamide. Schematic illustrations of the fabricated NH and ND array coated with MAPbI${}_{3}$ thin film are displayed in Figure 1a,b, respectively.

The thickness of the synthesized MAPbI${}_{3}$ films were characterized using a variable angle spectroscopic ellipsometer (J.M. Woollam, Lincoln, NE, USA, M-2000). The reflectance spectra were characterized using an inverted microscope (Nikon Instruments, Melville, NY, USA, Eclipse Ti) with a small numerical aperture objective (10×, NA = 0.3) to mimic normal incidence. A randomly polarized broadband light source was used to illuminate the sample. The reflected light from the samples was coupled into a spectrograph (Princeton Instruments, Acton, MA, USA, Acton SpectraPro SP-2300) and was detected using a charge-coupled device camera (Princeton Instruments, Acton, MA, USA, Pixis 100). A piece of flat silicon wafer was used as a calibration reference during the characterization.

Apart from experimentally characterizing the absorptance spectra, simulations are also performed using FDTD method. A commerical-grade 3D electromagnetic simulator from Lumerical Inc. (Vancouver, BC, Canada) was used [37]. Anti-symmetric and symmetric boundary conditions were used in the lateral directions while perfectly matched layer boundary conditions were used in the vertical directions. The real and imaginary parts of refractive indices for gold and MAPbI${}_{3}$ were obtained from literature [38,39]. Considering the balance between computational cost and accuracy for different structures, the mesh size varied among 4 nm × 4 nm × 2 nm, 4 nm × 4 nm × 1 nm and 3 nm × 3 nm × 2 nm in the Cartesian coordinates.

## 3. Results and Discussion

**Figure 2:**
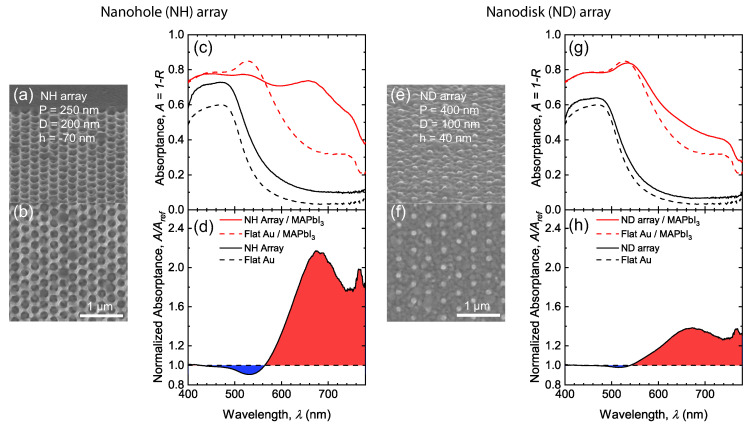
(**a**,**b**,**e**,**f**) display the tilted-view (**a**,**e**) and top-view (**b**,**f**) SEM micrographs of the fabricated nanohole (NH) array with *P* = 250 nm, *D* = 200 nm, *h* = −70 nm (**a**,**b**) and nanodisk (ND) array with *P* = 400 nm, *D* = 100 nm, *h* = 40 nm (**e**,**f**). (**c**,**g**) The measured absorptance spectra A=1−R for nanostructured (solid lines) or flat (dashed lines) gold with (red lines) or without (black lines) 110nm-thick MAPbI${}_{3}$ thin-film. (**d**,**h**) show the absorptance spectra for 110nm-thick MAPbI${}_{3}$ thin-films on nanostructured gold normalized to that of equal-thickness MAPbI${}_{3}$ on flat gold. The red filling ($A/A_{ref}>1.0$) represents absorption enhancement while the blue filling ($A/A_{ref}<1.0$) represents absorption suppression.

Figure 2a,b,e,f show the tilted and top-view scanning electron microscope (SEM) micrographs of example NH and ND arrays prior to the MAPbI${}_{3}$ synthesis. To investigate the absorption enhancement brought by the plasmonic nanostructure, the corresponding reflectance spectra R(λ) were measured, and the measured absorptance spectra A(λ)=1−R(λ) are reported in Figure 2c,g. Absorptance of nanostructured gold surface (black solid lines) shows a similar spectral shape as that of flat gold surface (black dashed lines) for both NH and ND arrays, while the NH array exhibits relatively higher absorption. This is because a portion of the scattered light by plasmonic concentrators is not collected by the finite NA of the objective lens, therefore a lower measured reflectance (i.e., higher absorption). By contrast, ND array in Figure 2e,f has smaller feature size and larger array pitch. Therefore, a smaller portion of light is scattered and a higher portion of reflected light is collected by the objective (i.e., lower absorption). The scattered light can be efficiently absorbed by adding a layer of MAPbI${}_{3}$ on the top. After being coated with a 110 nm-thick MAPbI${}_{3}$ film, the absorptance spectrum of the flat-interface structure (red dashed lines) shows a peak at wavelength λ = 526 nm. This results from the Fabry–Pérot cavity effect supported by the perovskite thin-film. The absorption decreases with increasing wavelength because the perovskite layer no longer supports any Fabry–Pérot cavity mode and the material has weaker absorbing property at longer wavelength. With NH or ND nanostructures (red solid lines), the absorption gets significantly enhanced at longer wavelengths, as is shown as red fillings in Figure 2d,h. For NH array, the spectral peak at λ = 526 nm gets weaker. This is because the structure disrupts the interference in the film [36]. In contrast, ND array still preserves that spectral peak due to its smaller feature size and lower nanostructure concentration. The absorptance spectra of perovskite/nanostructured gold are normalized to that of perovskite/flat gold and are displayed in Figure 2d,h. The blue filling at lower wavelengths represents the suppression of Fabry–Pérot cavity mode while the red filling represents the absorption enhancement brought by the plasmonic nanostructure. It is obvious that the example NH could not preserve the cavity effect as well as ND array (larger blue filling area), but the greater absorption enhancement by the plasmonic effects at longer wavelengths compensates for the loss at lower wavelengths (larger red filling area). As a comparison, for the example ND array, both blue and red filling areas are much smaller in Figure 2h, indicating negligible suppression at short wavelengths but also less significant enhancement at longer wavelengths.

**Figure 3:**
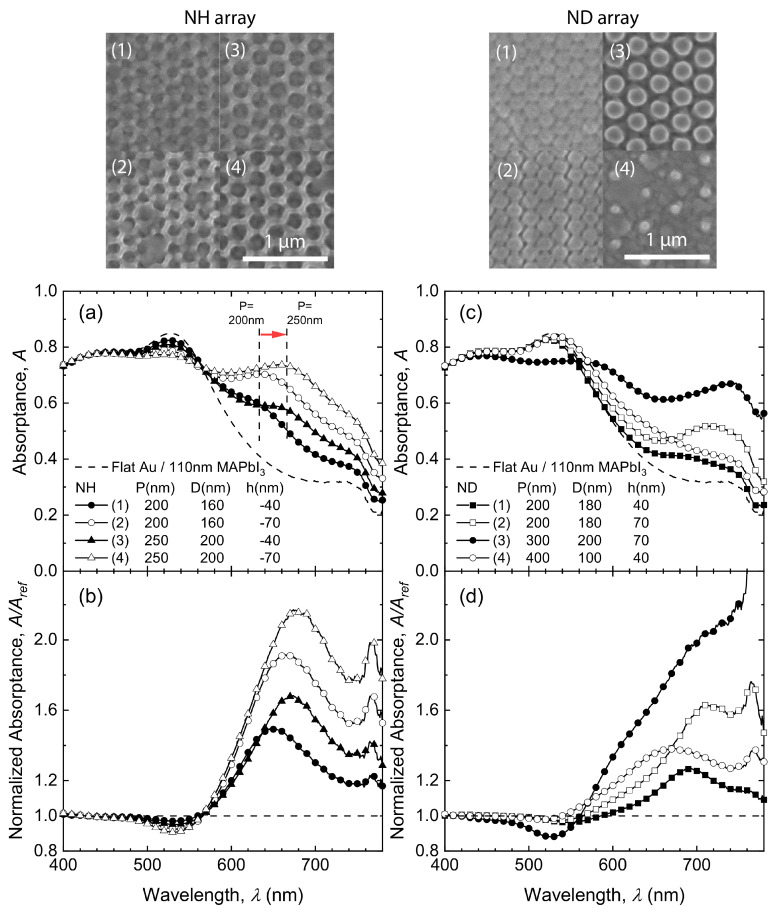
Top-view SEM micrographs (top), experimental absorptance spectra (**a**,**c**) and normalized absorptance spectra (**b**,**d**) for 110nm-thick MAPbI${}_{3}$ on flat (dashed lines) and nanostructured (solid lines with symbols) gold NH (left column) and ND (right column) arrays. The vertical dashed lines in (**a**) show the absorption spectral redshift as a function of pitch *P* (increasing from 200 nm to 250 nm).

A variety of nanostructured gold surfaces (NH or ND arrays) were fabricated based on optimal design considerations [36]. The top row of Figure 3 shows the SEM micrographs of the NH (left) and ND arrays (right). The measured and normalized absorptance spectra of these NH and ND arrays are shown in Figure 3a–d. For NH arrays (left column), as pitch *P* increases from 200 nm (circle symbols) to 250 nm (triangle symbols), the spectral peaks redshift from λ = 632 nm to λ = 666 nm. This results from the pitch-dependent collective surface plasmon polariton (SPP) resonances in the hexagonal lattice [36,40]. In addition, with hole depth increasing from 40 nm (solid symbols) to 70 nm (open symbols), the Fabry–Pérot cavity mode gets disrupted, resulting in a relatively lower absorption at λ = 526 nm, but there is more significant absorption enhancement at longer wavelengths. A similar behavior is also observed in ND arrays: structures suppressing the Fabry–Pérot thin-film interference effects show stronger absorption enhancement near the MAPbI${}_{3}$ band edge.

**Figure 4:**
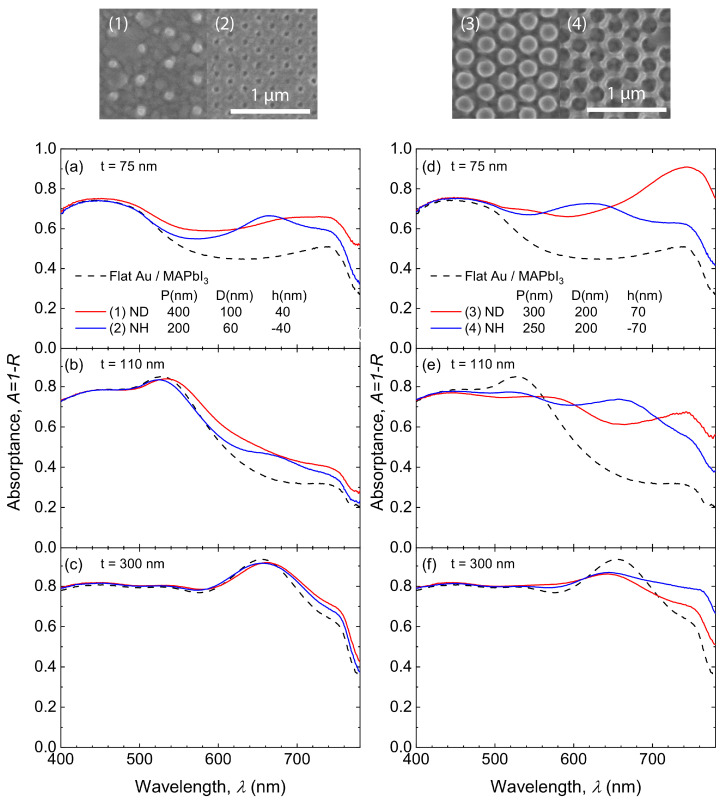
Measured absorptance spectra of flat (dashed lines) and nanostructured (solid lines) gold surfaces coated with varying-thickness MAPbI${}_{3}$ with *t* = 75 nm (**a**,**d**); 110 nm (**b**,**e**); 300 nm (**c**,**f**). The left (right) column corresponds to nanostructured gold surfaces with low (high) surface density of optical scatterers. The top panels show top-view SEM micrographs of gold surfaces with low (1–2) and high-concentration (3–4) ND or NH arrays.

The absorption enhancement with different perovskite layer thicknesses is also investigated experimentally. MAPbI${}_{3}$ layers with different thicknesses (*t* = 75, 110, 300 nm) were synthesized on the same nanostructure and the corresponding absorptance spectra were characterized and displayed in Figure 4. The spectral peaks and dips in the flat-interface cases (dashed lines) again result from the Fabry–Pérot cavity modes supported by different perovskite thicknesses at different wavelengths. The SEM micrographs of nanostructured surfaces are displayed in the top panels. Low-density and small-feature-size nanostructures and the corresponding absorptance spectra are shown in the left column. For different thicknesses *t*, these two structures can preserve the Fabry–Pérot cavity modes, as well as bring additional absorption enhancement. However, the absorption enhancement becomes much less significant as *t* increases to 300 nm, because most of the incident light is absorbed by the perovskite material before interacting with the plasmonic concentrators. For NH or ND arrays with larger feature sizes and higher concentration (right column), Fabry–Pérot cavity effects are significantly suppressed at the resonant wavelengths. In addition, both high-density and large-feature-size arrays exhibit stronger absorption enhancement at other wavelength ranges comparing to the low-density and small-feature-size arrays. Moreover, obvious absorption enhancement is observed even when *t* increases to 300 nm.

**Figure 5:**
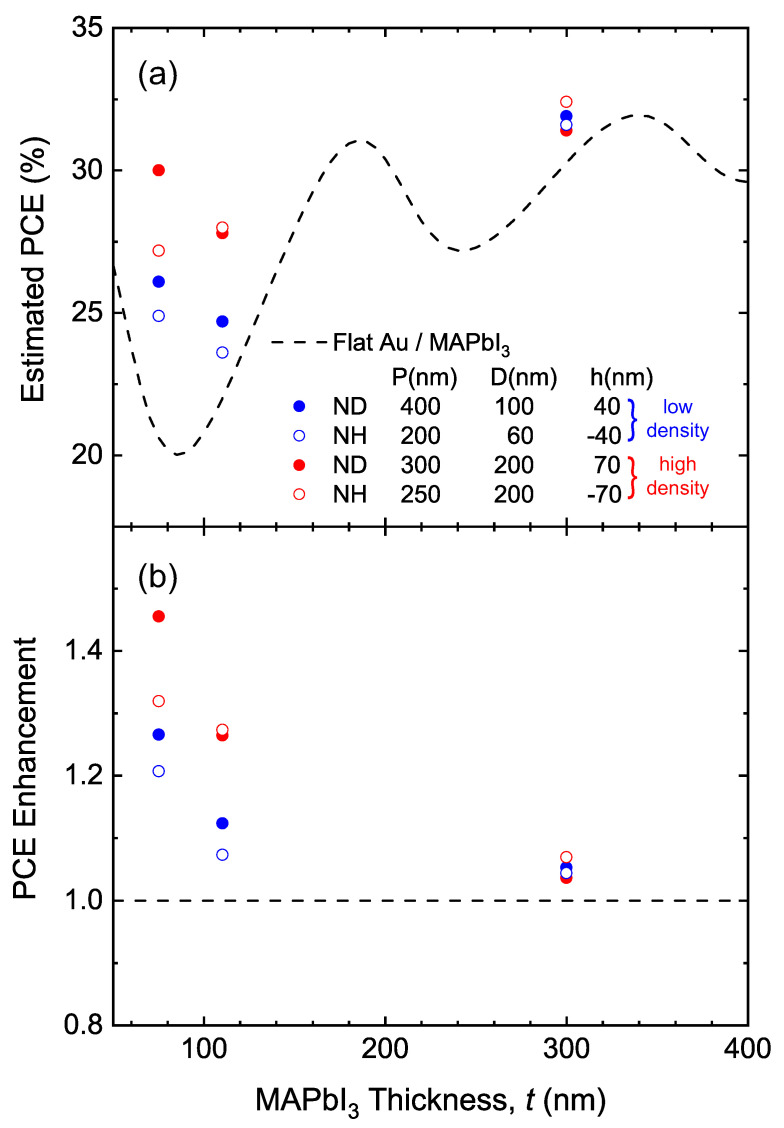
(**a**) Power conversion efficiency (PCE) and (**b**) PCE enhancement estimated from experimental absorptance spectra for varying MAPbI${}_{3}$ thickness values, *t* = 75, 110 and 300 nm with low (blue symbols) and high (red symbols) surface density of optical scatterers in NH (open symbols) and ND arrays (solid symbols). The dashed lines represent the PCE of flat-interface structures as a function of MAPbI${}_{3}$ thickness. A strong PCE enhancement is clearly demonstrated for high-density nanostructures at *t* = 75 and 110 nm (red symbols).

To further evaluate how the plasmonic effects and absorption enhancement will affect the overall performance of real photovoltaic devices, the PCE can be obtained using the measured absorptance spectra for different active layer thicknesses. With the detailed balance assumption, PCE can be expressed as

(1) $$\mathrm{PCE}=\frac{\int_{\lambda <\lambda_{g}}^{}\mathrm{AM}1.5(\lambda )\frac{2\pi \lambda }{\hbar c}A\left(\lambda \right)E_{g}d\lambda }{1\;sun}$$

where $\lambda_{g}$ is the bandgap wavelength of MAPbI${}_{3}$, AM1.5(λ) is the wavelength-dependent air mass 1.5 solar radiation spectrum, *ℏ* is the reduced Planck constant and 1sun is the 1000 W/m${}^{2}$ incident solar power density [41]. The experimentally measured absorptance spectra includes the absorption in both gold and MAPbI${}_{3}$, but the portion absorbed by gold is relatively small according to previously reported study [36]. For NH and ND arrays in Figure 4 coated with multiple active layer thicknesses, the calculated PCE and PCE enhancement is shown in Figure 5.

The PCE of flat structure PCE${}_{flat}$(t) (dashed line) is obtained using the multilayer interference model. For all three thicknesses (*t* = 75, 110 and 300 nm), all four nanostructure arrays exhibit PCE enhancement. Specifically, for active layer thickness *t* = 75 nm, high-density NH and ND arrays (red symbols) increase the PCE by 26.6% and 45.5%, respectively.

As a comparison, low-density nanostructure arrays (blue symbols) do not enhance the PCE as much. As the thickness increases to 300 nm, the PCE enhancement becomes much less significant due to the weaker plasmonic effect. The performance difference narrows between arrays of different concentration and feature sizes, enhancing the PCE by 3.7–7.0%.

As demonstrated in the experiment, plasmonic nanostructures with various geometry parameters exhibit different absorption enhancement behavior. Therefore, FDTD simulations were performed to further investigate the influence of different geometry parameters on the absorption enhancement.

**Figure 6:**
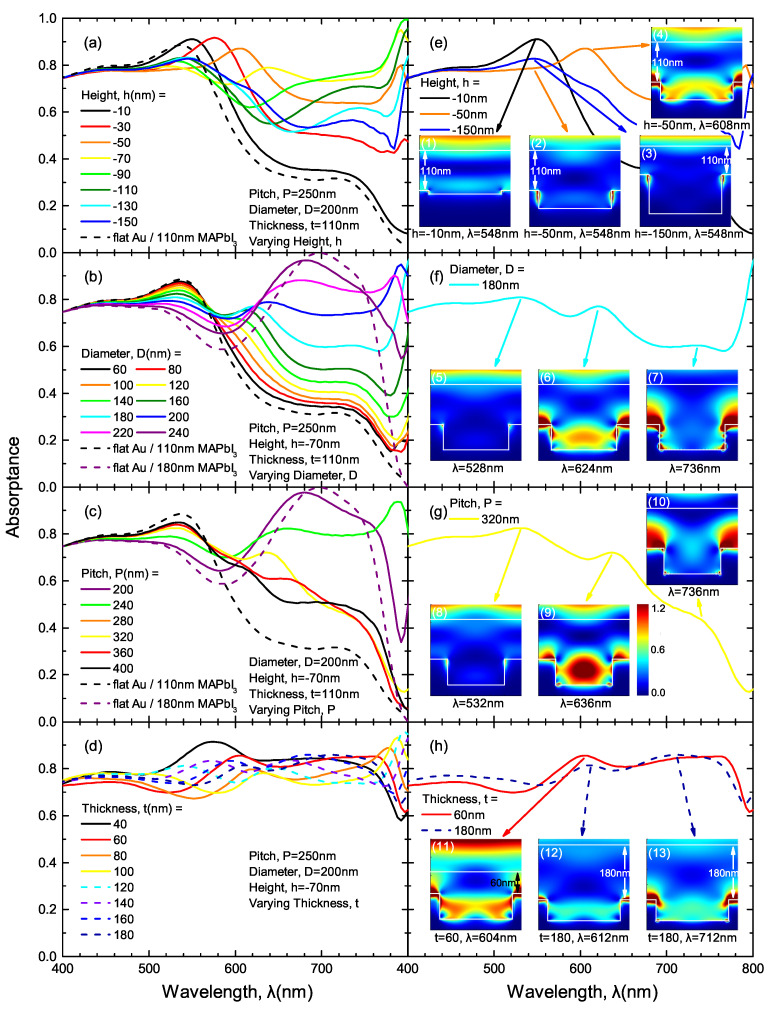
(**a**–**d**) Simulated absorptance spectra of representative NH arrays coated with a 110 nm-thick MAPbI${}_{3}$ film. Each of the geometry parameters (i.e., height, *h*, diameter, *D*, pitch, *P* and MAPbI${}_{3}$ thickness, *t*) is individually varied while keeping the others constant. (**e**–**h**) Representative absorptance spectra from (**a**–**d**) showing spectral peaks of interest. The color maps reported as insets show cross sections of electric field intensity simulated at each corresponding spectral peak wavelength by using 3D finite-difference time-domain (FDTD).

Figure 6a–d display the simulated absorptance spectra of an example NH array (*P* = 250 nm, *D* = 200 nm, *h* = −70 nm) coated with 110 nm-thick MAPbI${}_{3}$ film. In the left column, each of the four parameters are individually varied, meanwhile the other three parameters are kept fixed. When each parameter is varied, specific spectra with spectral peaks of interest are selected and respectively exhibited in Figure 6e–h and the insets show the electric field intensity distribution at the peak wavelengths.

First, the role of NH depth |h| is investigated. It is noticeable that the spectral peak at λ = 545 nm redshifts when |h| increases from 10 nm (black solid line) to 70 nm (yellow line). This peak results from the shallow hole, not disrupting the Fabry–Pérot effect (the bright horizontal band in inset (1)). When |h| increases to 50 nm, the peak redshifts due to a localized surface plasmon resonance (LSPR) arising inside the hole (inset (2) in Figure 6e). When |h| further increases to 150 nm, the constructive interference near the MAPbI${}_{3}$/gold interface is restored, but is not as strong (faint horizontal band in inset (3) in Figure 6e).

Figure 6b,c respectively demonstrate the absorptance spectra change with varying pitch *P* and diameter *D*. The black and purple dashed lines correspond to absorptance spectra of 110 nm and (110+|h|) nm-thick MAPbI${}_{3}$ film on flat gold, respectively. With small feature sizes (small diameter *D*) or low NH concentration (large pitch *P*), the absorptance spectra are similar to that of 110 nm-thick MAPbI${}_{3}$ on flat gold (black dashed lines). As the NH size or concentration increases, the absorptance spectral shape starts to shift towards that of 180 nm-thick MAPbI${}_{3}$ on flat gold (purple dashed lines), which results from the Fabry–Pérot mode between the bottom of the holes and top of MAPbI${}_{3}$ film. In addition, another peak near 630 nm shows up in some structures. Insets (6, 9) in Figure 6f,g show that these spectral peaks attribute to the LSPR inside the NH structure. Moreover, an additional spectral peak is observed at λ = 736 nm. These peaks results from the LSPR between neighboring NH structures, as is shown in insets (7, 10) in Figure 6f,g. Figure 6d demonstrates the thickness dependence of the absorptance spectra. With increasing thickness, the multiple peaks corresponds to the LSPR inside the holes or between neighboring holes. In addition, the resonances gets weaker as *t* increases due to less light–matter interaction.

**Figure 7:**
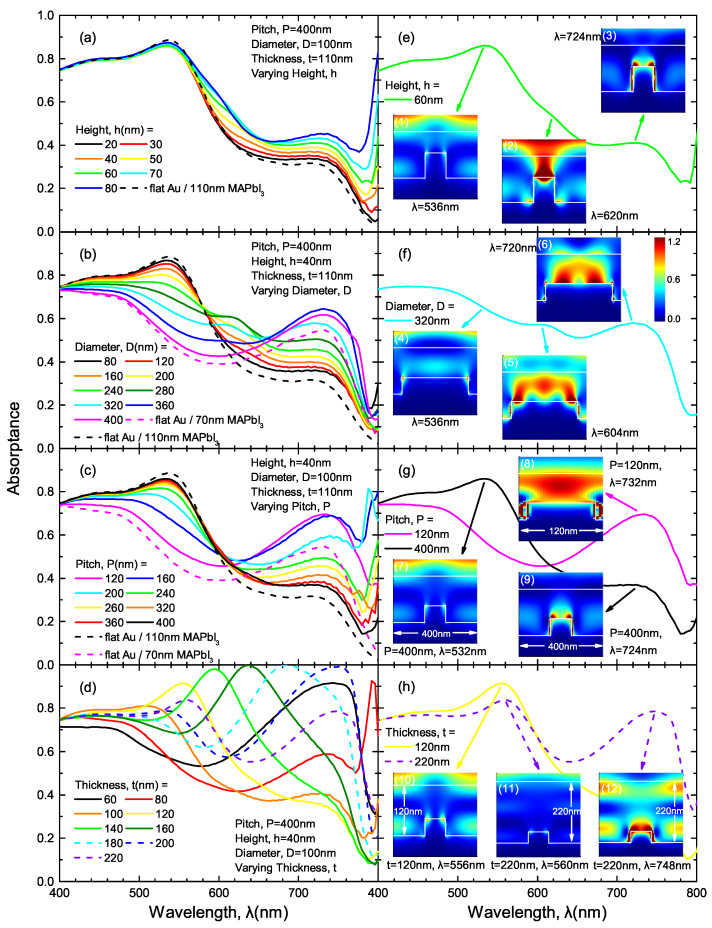
(**a**–**d**) Simulated absorptance spectra of representative ND arrays coated with a 110 nm-thick MAPbI${}_{3}$ film. The geometry parameters (i.e., height, *h*, diameter, *D*, pitch, *P* and MAPbI${}_{3}$ thickness, *t*) are individually varied while the other three parameters are kept constant; (**e**–**h**) Representative absorptance spectra from (**a**–**d**) showing spectral peaks of interest. The color maps reported as insets show cross sections of electric field intensity simulated at each corresponding spectral peak wavelength by using 3D FDTD.

Likewise, the role of the ND geometry parameters in absorption enhancement is also investigated. Figure 7 shows the simulated absorptance spectra of an example ND array (*P* = 400 nm, *D* = 100 nm, *h* = 40 nm) coated with MAPbI${}_{3}$ thin film of *t* = 110 nm.

While varying *h* (Figure 7a), the absorptance spectra shape mainly follows that of flat-interface structure (dashed black line), showing peaks at λ = 530 nm, which corresponds to the Fabry–Pérot effect of 110 nm-thick MAPbI${}_{3}$ on gold. Insets (1, 3) in Figure 7e show a discontinuous horizontal band, indicating that the existence of ND disrupts the cavity effect, but the spectral peaks still persists. Additionally, the ND also brings additional plasmonic resonances, as is shown in inset (3), enhancing the absorption with increasing height. Moreover, for a specific structure (*h* = 60 nm), another resonance is observed on the top of the disk structure, as is shown in inset (2) in Figure 7e.

The diameter *D* and array pitch *P* play a similar role in ND arrays: smaller feature sizes and low concentration preserve cavity effects, but also contribute to less plasmonic effect, as is shown in Figure 7b,c. For *D* = 320 nm (Figure 7f), specifically, the suppression of Fabry–Pérot effect is observed (inset (4) in Figure 7f). Similar to NH arrays, high-density (small *P*) ND arrays also suppress the cavity effects, while contributing to significant plasmonic effects (insets (7–9) in Figure 7g). In addition, multiple orders of localized standing-wave surface plasmonic resonances are observed on the top surface of the ND structure, as are reported in insets (5, 6) in Figure 7f.

Figure 7d shows the absorptance spectra of structures with varying MAPbI${}_{3}$ thickness *t* coated on the ND arrays. Due to the small ND size, as well as the relatively large array pitch, the spectra peaks generally result from the Fabry–Pérot cavity effects (horizontal bands in insets (10–12) in Figure 7h). In spite of the existence of the ND structure, the modes still persist.

**Figure 8:**
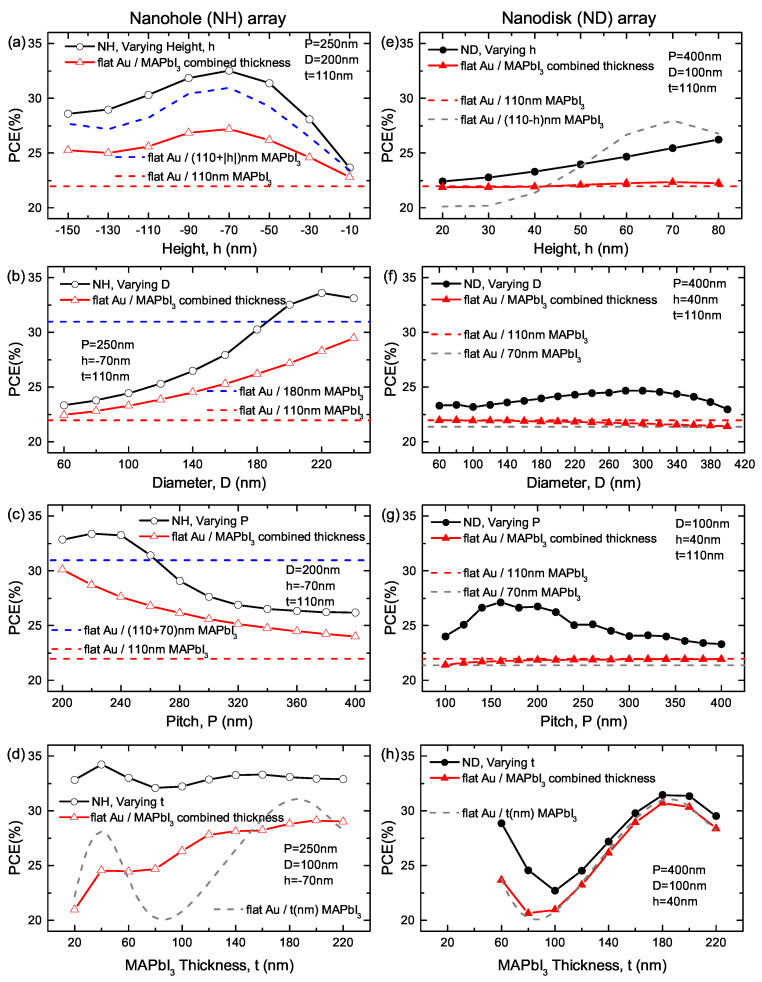
Calculated PCE for NH (**a**–**d**) and ND (**e**–**h**) arrays as a function of varying geometry parameters (i.e., height, *h*, diameter, *D*, array pitch, *P* and MAPbI${}_{3}$ thickness, *t*). Dashed lines show the PCE of flat structures with *t* = 110 nm or 110±|h| nm, while red triangle lines show the PCE of flat structures with a combined thickness that accounts for the presence of two effective Fabry–Pérot cavities in the nanostructured structure.

The PCE is calculated based on the previous simulation results, and is reported as black circle lines in Figure 8. The PCE of NH and ND structure are respectively displayed in the left and right column. As is expected, when coated with 110nm-thick MAPbI${}_{3}$, most of the structures with nanostructured surfaces exhibit significantly enhanced PCE (black circles) compared to their flat-interface counterparts (red dashed lines). It is worth noting that NH structures are adding extra perovskite material volume to the 110 nm-thick film, while ND structures have less perovskite material than the flat structure. To take this into account, two additional references are exhibited in Figure 8: (1) the blue dashed lines in Figure 8a–c and gray dashed lines in Figure 8e–g respectively show PCE${}_{flat}$(t = 110±|h| nm) for NH and ND arrays; (2) the red triangle lines represent the PCE of flat structures with a combined thickness that accounts for the presence of two effective Fabry–Pérot cavities, which can be respectively expressed as

(2) $$\mathrm{PCE}{}_{combined}=\frac{\pi D^{2}}{2\sqrt{3}P^{2}}\mathrm{PCE}{}_{flat}(t=110\pm |h|\mathrm{nm})+\left(1-\frac{\pi D^{2}}{2\sqrt{3}P^{2}}\right)\mathrm{PCE}{}_{flat}\left(t=110\mathrm{nm}\right)$$

where the plus (minus) sign refers to NH (ND) arrays. For most of the structures, the nanostructured-surface PCE is greater than PCE${}_{combined}$, which further proves that the plasmonic effects improve the solar cell performance. In addition, the difference between these two PCEs generally increases with increasing *D* and decreasing *P*, i.e., the cell performance benefits more from high-density and large-size nanostructures. In addition, with small thickness *t*, a larger portion of light penetrates the perovskite active layer, interacting strongly with the nanostructure array, and therefore also results in a larger difference.

## 4. Conclusions

In conclusion, various plasmonic nanostructure arrays are experimentally fabricated on gold surfaces coated with perovskite films with varying thickness and their optical absorption properties are theoretically calculated by using 3D FDTD simulations. It is shown that, although thin-film interference effects can be negatively affected by the presence of nanoscatterers, overall, the plasmonic concentrators allow for significant absorption enhancement as the result of a combination of physical effects, i.e., (1) localized optical resonances (including surface plasmon resonances); (2) plasmonic modes in vertical cavities (such as the space within nanoholes); (3) in-plane constructive interference of surface plasmon polaritons that propagate along the metal/dielectric interface. Structures with higher surface density of nanoscattereres and with reduced film thickness generally show larger absorption and calculated PCE enhancements. The experimental results are supported by 3D FDTD simulations that help identify the individual physical effects and elucidate their interplay in redistributing the incident field intensity, which in turns determine the observed absorption and the calculated PCE enhancements.

## References

[1] Huang J., Yuan Y., Shao Y., Yan Y. (2017). Understanding the physical properties of hybrid perovskites for photovoltaic applications. Nat. Rev. Mater..

[2] Dong Q., Fang Y., Shao Y., Mulligan P., Qiu J., Cao L., Huang J. (2015). Electron-hole diffusion lengths > 175 *μ*m in solution-grown CH_3_NH_3_PbI_3_ single crystals. Science.

[3] Yang S., Fu W., Zhang Z., Chen H., Li C.Z. (2017). Recent advances in perovskite solar cells: Efficiency, stability and lead-free perovskite. J. Mater. Chem. A.

[4] Elumalai N.K., Mahmud M.A., Wang D., Uddin A. (2016). Perovskite solar cells: Progress and advancements. Energies.

[5] Chen M., Ju M.G., Carl A.D., Zong Y., Grimm R.L., Gu J., Zeng X.C., Zhou Y., Padture N.P. (2018). Cesium titanium (IV) bromide thin films based stable lead-free perovskite solar cells. Joule.

[6] Song Z., Watthage S.C., Phillips A.B., Heben M.J. (2016). Pathways toward high-performance perovskite solar cells: Review of recent advances in organo-metal halide perovskites for photovoltaic applications. J. Photon. Energy.

[7] Zhou Y., Zhou Z., Chen M., Zong Y., Huang J., Pang S., Padture N.P. (2016). Doping and alloying for improved perovskite solar cells. J. Mater. Chem. A.

[8] Best Research–Cell Efficiencies. https://www.nrel.gov/pv/assets/pdfs/best-research-cell-efficiencies.20200406.pdf.

[9] Ferry V.E., Sweatlock L.A., Pacifici D., Atwater H.A. (2008). Plasmonic nanostructure design for efficient light coupling into solar cells. Nano Lett..

[10] Atwater H.A., Polman A. (2011). Plasmonics for improved photovoltaic devices. Materials For Sustainable Energy: A Collection of Peer-Reviewed Research and Review Articles from Nature Publishing Group.

[11] Polman A., Atwater H.A. (2012). Photonic design principles for ultrahigh-efficiency photovoltaics. Nat. Mater..

[12] Gan Q., Bartoli F.J., Kafafi Z.H. (2013). Plasmonic-enhanced organic photovoltaics: Breaking the 10% efficiency barrier. Adv. Mater..

[13] Yang J., You J., Chen C.C., Hsu W.C., Tan H.R., Zhang X.W., Hong Z., Yang Y. (2011). Plasmonic polymer tandem solar cell. ACS Nano.

[14] Ding I.K., Zhu J., Cai W., Moon S.J., Cai N., Wang P., Zakeeruddin S.M., Grätzel M., Brongersma M.L., Cui Y. (2011). Plasmonic dye-sensitized solar cells. Adv. Energy Mater..

[15] Spinelli P., Ferry V., Van de Groep J., Van Lare M., Verschuuren M., Schropp R., Atwater H., Polman A. (2012). Plasmonic light trapping in thin-film Si solar cells. J. Opt..

[16] Jiménez-Solano A., Carretero-Palacios S., Míguez H. (2018). Absorption enhancement in methylammonium lead iodide perovskite solar cells with embedded arrays of dielectric particles. Opt. Express.

[17] Miranda-Muñoz J.M., Carretero-Palacios S., Jiménez-Solano A., Li Y., Lozano G., Míguez H. (2016). Efficient bifacial dye-sensitized solar cells through disorder by design. J. Mater. Chem. A.

[18] Carretero-Palacios S., Jiménez-Solano A., Míguez H. (2016). Plasmonic nanoparticles as light-harvesting enhancers in perovskite solar cells: A user’s guide. ACS Energy Lett..

[19] Carretero-Palacios S., Calvo M.E., Míguez H. (2015). Absorption enhancement in organic–inorganic halide perovskite films with embedded plasmonic gold nanoparticles. J. Phys. Chem. C.

[20] Kang S.M., Jang S., Lee J.K., Yoon J., Yoo D.E., Lee J.W., Choi M., Park N.G. (2016). Moth-Eye TiO_2_ Layer for Improving Light Harvesting Efficiency in Perovskite Solar Cells. Small.

[21] Mali S.S., Shim C.S., Kim H., Patil P.S., Hong C.K. (2016). In situ processed gold nanoparticle-embedded TiO_2_ nanofibers enabling plasmonic perovskite solar cells to exceed 14% conversion efficiency. Nanoscale.

[22] Ghahremanirad E., Olyaee S., Hedayati M. (2019). The Influence of Embedded Plasmonic Nanostructures on the Optical Absorption of Perovskite Solar Cells. Photonics.

[23] Mohsen A.A., Zahran M., Habib S., Allam N.K. (2020). Refractory plasmonics enabling 20% efficient lead-free perovskite solar cells. Sci. Rep..

[24] Deng W., Yuan Z., Liu S., Yang Z., Li J., Wang E., Wang X., Li J. (2019). Plasmonic enhancement for high-efficiency planar heterojunction perovskite solar cells. J. Power Sour..

[25] Wang B., Zhu X., Li S., Chen M., Liu N., Yang H., Ran M., Lu H., Yang Y. (2019). Enhancing the Photovoltaic Performance of Perovskite Solar Cells Using Plasmonic Au@ Pt@ Au Core-Shell Nanoparticles. Nanomaterials.

[26] Yao K., Zhong H., Liu Z., Xiong M., Leng S., Zhang J., Xu Y.X., Wang W., Zhou L., Huang H. (2019). Plasmonic Metal Nanoparticles with Core–Bishell Structure for High-Performance Organic and Perovskite Solar Cells. ACS Nano.

[27] Chen L.C., Tien C.H., Lee K.L., Kao Y.T. (2020). Efficiency Improvement of MAPbI_3_ Perovskite Solar Cells Based on a CsPbBr_3_ Quantum Dot/Au Nanoparticle Composite Plasmonic Light-Harvesting Layer. Energies.

[28] Lu Z., Pan X., Ma Y., Li Y., Zheng L., Zhang D., Xu Q., Chen Z., Wang S., Qu B. (2015). Plasmonic-enhanced perovskite solar cells using alloy popcorn nanoparticles. RSC Adv..

[29] Zhang W., Saliba M., Stranks S.D., Sun Y., Shi X., Wiesner U., Snaith H.J. (2013). Enhancement of perovskite-based solar cells employing core–shell metal nanoparticles. Nano Lett..

[30] Bayles A., Carretero-Palacios S., Calió L., Lozano G., Calvo M.E., Míguez H. (2020). Localized surface plasmon effects on the photophysics of perovskite thin films embedding metal nanoparticles. J. Mater. Chem. C.

[31] Ostfeld A., Pacifici D. (2011). Plasmonic concentrators for enhanced light absorption in ultrathin film organic photovoltaics. Appl. Phys. Lett..

[32] Mao J., Sha W.E., Zhang H., Ren X., Zhuang J., Roy V.A., Wong K.S., Choy W.C. (2017). Novel direct nanopatterning approach to fabricate periodically nanostructured perovskite for optoelectronic applications. Adv. Funct. Mater..

[33] Wang H., Haroldson R., Balachandran B., Zakhidov A., Sohal S., Chan J.Y., Zakhidov A., Hu W. (2016). Nanoimprinted perovskite nanograting photodetector with improved efficiency. ACS Nano.

[34] Long M., Chen Z., Zhang T., Xiao Y., Zeng X., Chen J., Yan K., Xu J. (2016). Ultrathin efficient perovskite solar cells employing a periodic structure of a composite hole conductor for elevated plasmonic light harvesting and hole collection. Nanoscale.

[35] Paetzold U.W., Qiu W., Finger F., Poortmans J., Cheyns D. (2015). Nanophotonic front electrodes for perovskite solar cells. Appl. Phys. Lett..

[36] Shen T., Siontas S., Pacifici D. (2018). Plasmon-enhanced thin-film perovskite solar cells. J. Phys. Chem. C.

[37] Lumerical Inc. https://www.lumerical.com/products/.

[38] Löper P., Stuckelberger M., Niesen B., Werner J., Filipic M., Moon S.J., Yum J.H., Topič M., De Wolf S., Ballif C. (2014). Complex refractive index spectra of CH_3_NH_3_PbI_3_ perovskite thin films determined by spectroscopic ellipsometry and spectrophotometry. J. Phys. Chem. Lett..

[39] Haynes W.M. (2014). CRC Handbook of Chemistry and Physics.

[40] Ng R.J., Goh X.M., Yang J.K. (2015). All-metal nanostructured substrates as subtractive color reflectors with near-perfect absorptance. Opt. Express.

[41] Würfel P., Würfel U. (2016). Physics of Solar Cells: From Basic Principles to Advanced Concepts.

